# Networks of reliable reputations and cooperation: a review

**DOI:** 10.1098/rstb.2020.0297

**Published:** 2021-11-22

**Authors:** Károly Takács, Jörg Gross, Martina Testori, Srebrenka Letina, Adam R. Kenny, Eleanor A. Power, Rafael P. M. Wittek

**Affiliations:** ^1^ The Institute for Analytical Sociology, Linköping University, 601 74 Norrköping, Sweden; ^2^ Computational Social Science—Research Center for Educational and Network Studies (CSS-RECENS), Centre for Social Sciences, Tóth Kálmán u. 4., 1097 Budapest, Hungary; ^3^ Institute of Psychology, Leiden University, Wassenaarseweg 52, 2333 AK, Leiden, The Netherlands; ^4^ Organization Sciences, Vrije Universiteit Amsterdam, De Boelelaan 1105, 1081 HV Amsterdam, The Netherlands; ^5^ Institute of Health and Wellbeing, MRC/CSO Social and Public Health Sciences Unit, University of Glasgow, Berkeley Square, 99 Berkeley Street, Glasgow G3 7HR, UK; ^6^ Institute of Cognitive and Evolutionary Anthropology, University of Oxford, 64 Banbury Road, Oxford OX2 6PN, UK; ^7^ Calleva Research Centre for Evolution and Human Sciences, Magdalen College, High Street, Oxford OX1 4AU, UK; ^8^ Department of Methodology, The London School of Economics and Political Science, Houghton Street, London WC2A 2AE, UK; ^9^ Department of Sociology, University of Groningen, Grote Rozenstraat 31, 9712 TG Groningen, The Netherlands

**Keywords:** cooperation, social networks, reputation, indirect reciprocity, intergroup relations, relational multiplexity

## Abstract

Reputation has been shown to provide an informal solution to the problem of cooperation in human societies. After reviewing models that connect reputations and cooperation, we address how reputation results from information exchange embedded in a social network that changes endogenously itself. Theoretical studies highlight that network topologies have different effects on the extent of cooperation, since they can foster or hinder the flow of reputational information. Subsequently, we review models and empirical studies that intend to grasp the coevolution of reputations, cooperation and social networks. We identify open questions in the literature concerning how networks affect the accuracy of reputations, the honesty of shared information and the spread of reputational information. Certain network topologies may facilitate biased beliefs and intergroup competition or in-group identity formation that could lead to high cooperation within but conflicts between different subgroups of a network. Our review covers theoretical, experimental and field studies across various disciplines that target these questions and could explain how the dynamics of interactions and reputations help or prevent the establishment and sustainability of cooperation in small- and large-scale societies.

This article is part of the theme issue ‘The language of cooperation: reputation and honest signalling’.

## 1. Introduction

Despite its obvious benefits, cooperation necessarily entails individual sacrifices, and so poses a fundamental puzzle for evolutionary theory, already alluded to by Darwin [1;2, pp. 5–8]. Many breakthroughs in evolution, however, have been achieved through cooperation and the formation of cooperative alliances [3]. For example, the transition from single-celled to multicellular organisms required the restriction of individual cell growth, cellular differentiation and the management of cheating—cooperation between cells to create a functioning organism [4–6]. Here, we focus our attention on cooperation in humans, as our species is particularly skilled in resolving cooperation problems and regularly cooperate with non-kin strangers [7,8]. It has been a major scientific achievement to realize and empirically demonstrate that humans are capable of solving the problem of cooperation through *reputation* [9–14]. Reputation can be defined as (shared) information about the qualities and attributes of an individual that also includes cooperativeness [15,16]. Reputational information can be used to condition behaviour towards an interaction partner. Furthermore, it can determine access to partner choice and reproduction as well as to material and to immaterial resources (e.g. to power). The benefits of having a ‘good’ reputation can deter individuals from defection and thereby foster the evolution of cooperation [17–19].

These processes do not take place in a social vacuum. Human interactions are complex, as they are embedded in social network structures. The local network ties that an individual has are important for solving the problem of cooperation for multiple reasons. First, the local network structure provides the context of social interdependencies, and large-scale cooperation is often scaled up from the successful establishment of local cooperation. Second, network ties provide the constraints of monitoring and controlling behaviour. Third, they are also the channels of communication. Communication is a device for coordination, but also for influence and persuasion towards doing the right thing for the larger group. The advanced communication capacities and social skills of humans allow us to disseminate third-party evaluations through *gossip* [14,20–22]. Gossip is the method through which one learns the reputations of others, and through which reputations are shared in the absence of direct experience and observation. These processes also sustain reputation-based cooperation in larger groups in which not every interaction can be monitored [23–25].

In short, network ties impact cooperation through multiple mechanisms. They can help the establishment of reputations that are aligned with group-beneficiary action but could potentially also be used to control the flow of, or strategically manipulate, reputational information [26].

After a short introduction to the problem of cooperation, we review work on the foundational elements of reputation-based cooperation in humans. We start from the simplest models that link reputation and cooperation, and then address the impact of networks on cooperation, before finally discussing more complex models of their coevolution. We conclude that the existing theoretical work on reputation and cooperation has not fully accounted for the possible complex interplay that emerges when social networks dynamically change as a function of gossip and reputation (e.g. [27]). While such dynamics pose many challenges, we argue that progressing the field towards investigating the interactions of reputation, gossip and network topology might help to overcome the remaining puzzles of how networks can ensure reliable reputation systems assisting the evolution of cooperation. Such a programme also promises explanations for when and why gossip and reputation dynamics have adverse effects on cooperation, by fostering dishonesty, strategic manipulation attempts, or by giving rise to parochial, group-bounded cooperation.

## 2. The problem of cooperation

Cooperation is defined as a costly action to benefit another individual, where the benefit *b* is higher than the cost *c* (with *b > c* and *c* > 0) [7]. What follows is that mutual cooperation leads to higher social welfare than mutual defection. In other words, working together creates synergies that exceed what individuals alone are capable of. Yet, individuals are even better off by reaping the benefits of cooperation without paying the cost of cooperation themselves. This temptation to free-ride on the cooperation of others introduces a ‘puzzle’: How can cooperation emerge, given the risk of exploitation and, concomitantly, the temptation to exploit?

Prominent theories in biology explain cooperation based on kinship ([28,29], cf. [30]) and reciprocal interactions [31–33]. While these theories apply to human cooperation as well, the remarkable extent of large-scale cooperation among non-kin observed in humans has shifted the attention to the role of reputation, communication, and social networks for sustaining cooperation.

## 3. Reputation as a mechanism for solving the problem of cooperation

Humans are able to observe the actions of others and exchange information. This allows the evolution of cooperation through mechanisms such as indirect reciprocity and reputation [12]. Communication and the ability to track reputation enable decision rules like ‘if someone told me that you cooperated with others in the past, I cooperate with you’. Cooperative decisions that are made conditional on transmitted information (i.e. ‘reputation’) also allow the extension of cooperation beyond dyadic relations. In a simple model, Nowak & Sigmund [11] showed that, if individuals base their decisions on the so-called image score of the interaction partner, simply operationalized as the number of times an individual helped others in the past, cooperators can avoid exploitation by identifying free-riders pre-emptively. Testing Nowak & Sigmund's model experimentally, Wedekind & Milinski [34] have shown that human participants are indeed sensitive to image scores and cooperate conditionally on whether the interaction partner has a good reputation (i.e. high image score).

Once good reputation pays off, individuals have an incentive to ‘invest’ in building it. Whether the expected benefits exceed the costs of investment depends on the size of the population, the reliability of the image score transmission, the number of future interactions and whether reputation provides a valid and reliable signal of cooperativeness in the first place [35]. The latter is open to exploitation as individuals may be able to increase their own reputation by buying ‘fake’ reputation (e.g. [36]). This means that reputation can be increased artificially without actually engaging in costly cooperation, enabling the exploitation of cooperators. Experimental results highlight how important the validity of reputation signals is and suggest that any reputation system has to mitigate the presence of adverse incentives to control and manipulate reputational information [37]. Recent studies have also shown that reputation scores can lose their ability to foster cooperation if they are assigned to groups rather than individuals or when cooperation takes place in groups rather than dyadic interactions [38,39].

Since the introduction of the ‘image score’ as a simplified concept of reputation, different rules on how to assign reputation based on past action have been proposed and analysed (see e.g. [40–46]). Not all rules can sustain cooperation. From all consensual attributions, only eight norms (the so-called leading eight) that determine proper action and assignment of good reputation have been shown to maintain cooperation while being resistant to mutation and observation error [44,45]. The joint properties of the leading eight norms are that (i) they assign good reputation for cooperation by actors with good reputation against others with good reputation (maintenance of cooperation); (ii) they assign bad reputation for defection against individuals with good reputation (identification of defectors); (iii) they maintain good reputation for actors with good reputation after defection against individuals with bad reputation (justified punishment); and (iv) they assign good reputation for actors with bad reputation if they cooperated with individuals with good reputation (forgiveness) [44,45].

Reputation is an important mechanism for the emergence of cooperation not only because it might be the basis of conditional cooperation, but also because it could be the basis of whom to learn from [47]. When individuals with high reputation discount the behavioural strategies of individuals with low reputation, cooperation is further enhanced, especially if discounting is based on absolute rather than on relative reputation [47].

Most importantly, reputation can also be the basis of partner choice [25,48–51]. Under ideal circumstances, reputational information allows predictions about the likely action of other agents in the population. Partner choice or ‘relational mobility’ allows cooperative agents to seek out partners that have a ‘good’ reputation and avoid agents with a ‘bad’ reputation. This creates competition for the attention of other cooperators in so-called biological markets in which agents compete to be selected as interaction partners [52–56]. In theory, if reputation provides a valid and unambiguous signal of an agent's cooperativeness, cooperators have a competitive advantage over defectors, since they can exclusively interact with each other and gain from the mutual benefits of cooperation.

Reputation, however, is not necessarily available publicly or shared universally [57–62]. While individuals may observe the actions of others, reputational information in humans is often also transmitted through gossip [63–66]. Gossip allows people to sustain cooperative behaviours through the spread of negative reputation and the fear of retaliation [17], as well as through the creation of coalitions and the exercise of ostracism [16,22,25,67,68]. Yet, gossip can also be used strategically by sharing false information to damage the reputation of others, raising the question of how groups evaluate gossip and detect false accusations and strategic misinformation [69–72]. In addition to strategic lies, gossip might entail a non-negligible degree of noise (that is, unintended errors). Gossip information can be wrongly communicated, impeding the correct transmission of reputational information and leading to mistakes in updating reputation of others [69,73]. Receivers may also wrongly interpret gossip information, likewise increasing the noise of reputational information [74,75]. Hess & Hagen [69] provided evidence that people evaluate the veracity of gossip information, for example, by assigning more credibility to gossip information coming from multiple, independent sources or taking into account the relationship between the gossiper and the gossip target, which can increase the reliability and validity of gossip and reduce strategic gossip lies and unintended errors. Such complexities are often not incorporated in formalized theoretical models, but play an important role in social groups, extend the function of reputation beyond simply identifying free-riders or cooperators, and introduce noise and strategic signalling, and psychological mechanisms to prevent the spread of false gossip.

## 4. Networks and cooperation

In large groups, people do not meet randomly, as assumed in some theoretical models for simplification. Human interactions are embedded in social networks that determine the likelihood with which two people meet, interact and exchange information. A logical implication is that the structure of a network could influence cooperation. Network topologies and features like how segmented, dense, close-knit or centralized a network is, might strongly facilitate or hinder the emergence of cooperation by constraining interactions or information transmission that might occur (table 1). In this section, we briefly review how networks can foster cooperation through mechanisms other than reputation.

**Table 1.** Key network concepts relevant for cooperation.

concept	definition/explanation	visual representation
network segmentation	the network can be partitioned into unconnected components; no influence on behaviour or on reputations is possible between the components	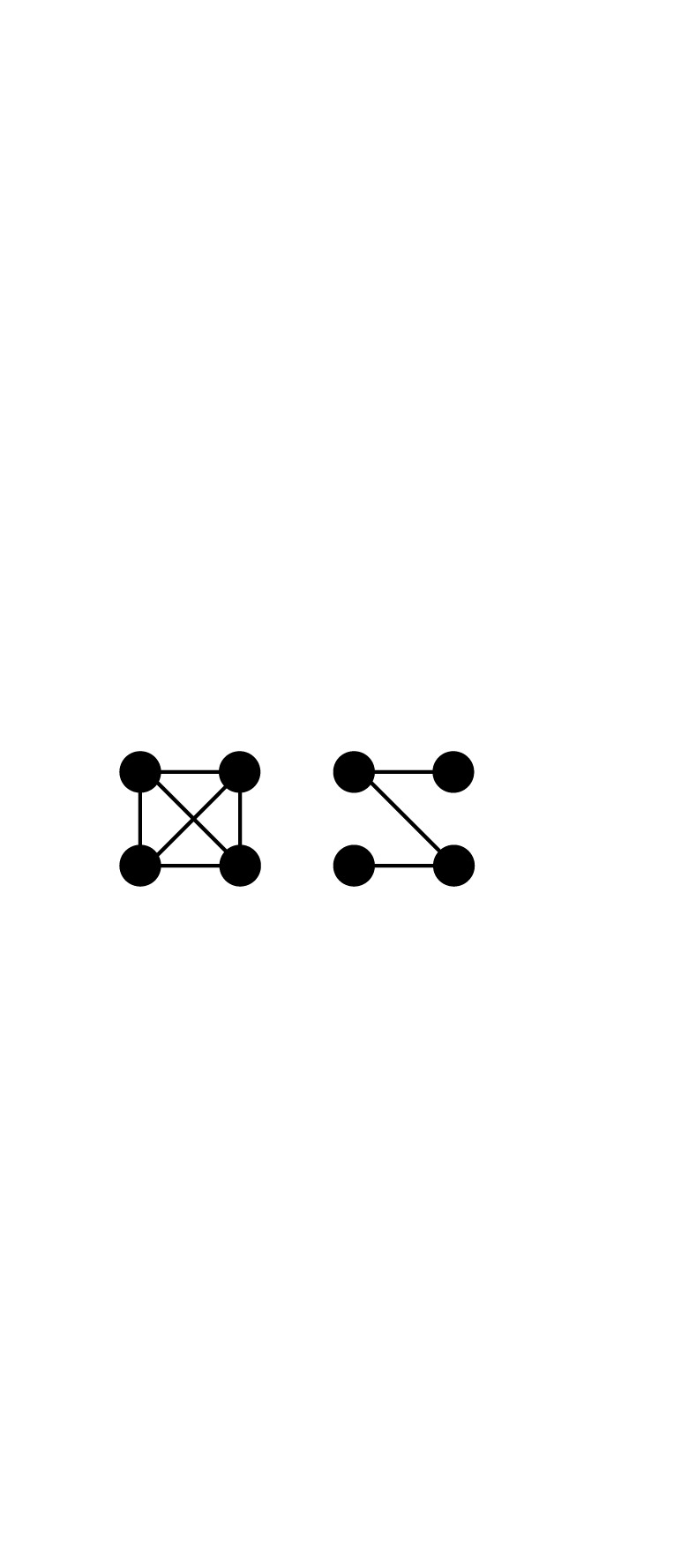
network clustering/modularity /segregation/assortativity of nodes/the small-world phenomenon	human networks are characterized by dense and cohesive communities in which individuals show a large amount of similarity with each other (indicated by node colour). These cohesive clusters (modules) are loosely connected with each other through bridging ties (dotted lines), resulting in shorter network distances and a small world [76,77].	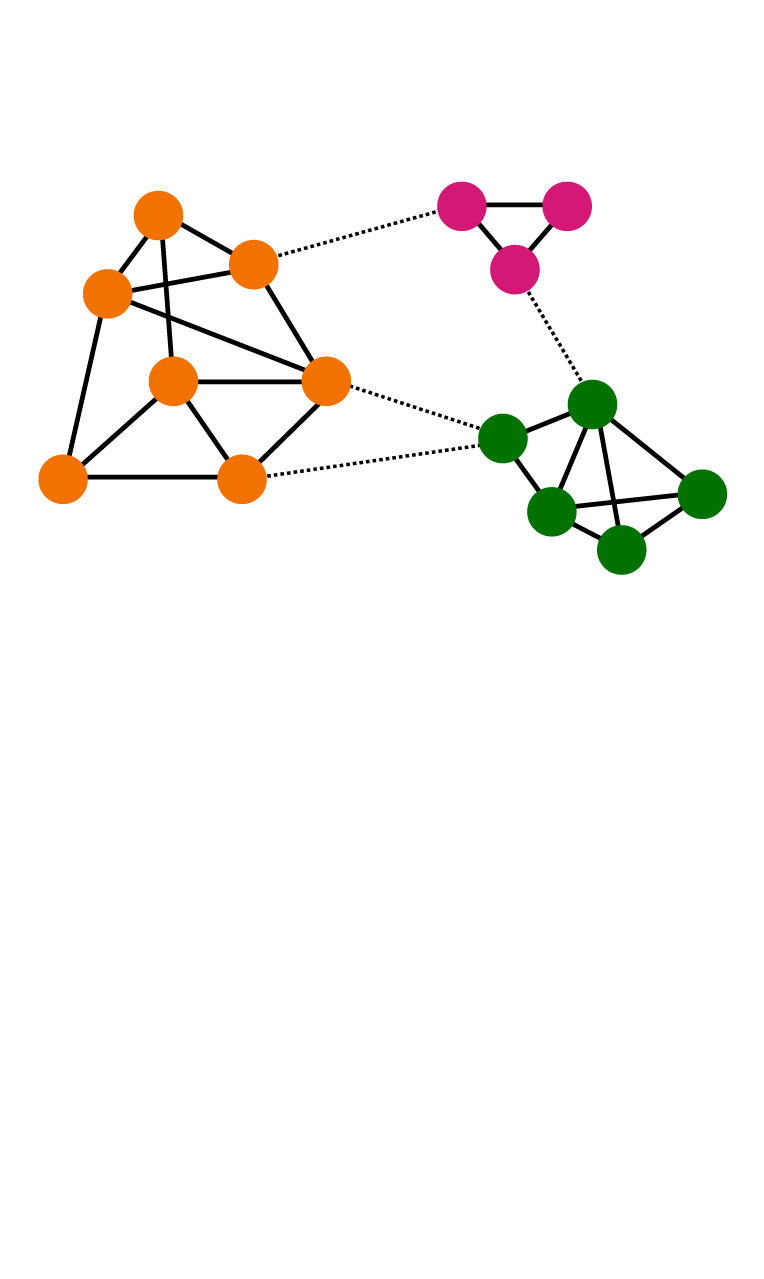
influence and selection/coevolution of networks and cooperation	the behavioural similarity of individuals in a community (cohesive subgraph) could be a result of social influence (assimilation, social learning) in informal relations or partner selection based on homophily [78,79]	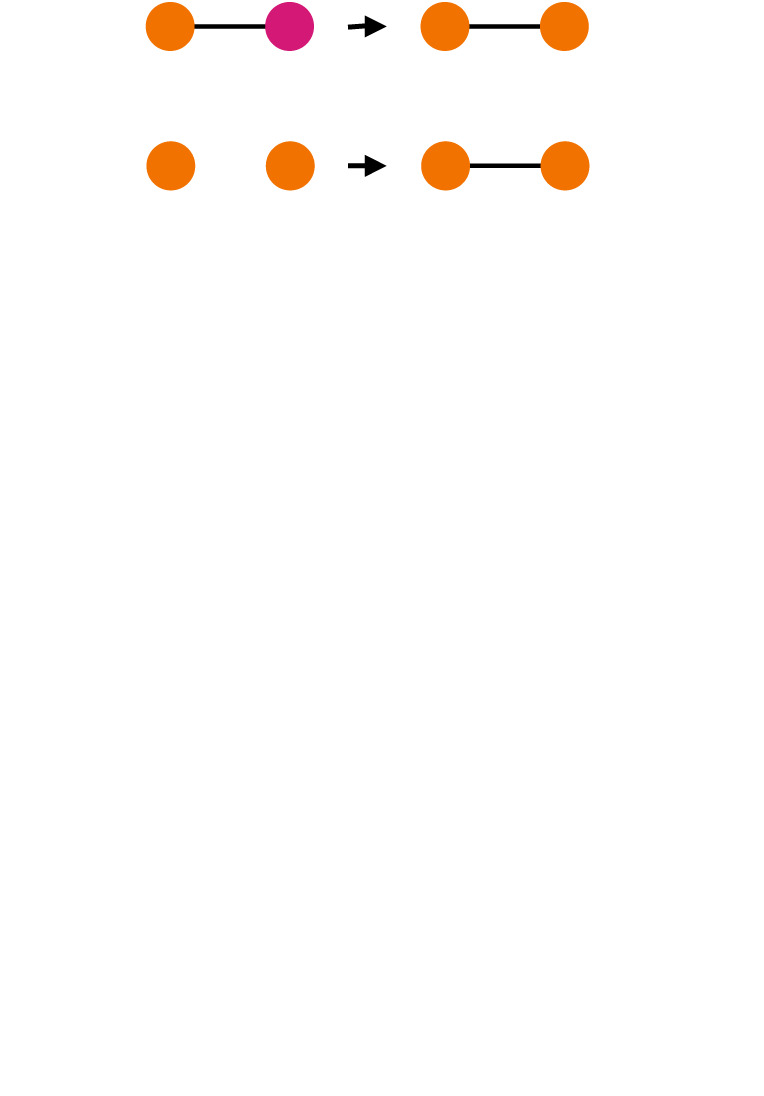
brokerage/betweenness/centrality/power/social control	individuals may be in a distinguished network position such that they connect otherwise unconnected others (brokers, red), most information flows through them (betweenness, yellow), can influence many others (centrality, purple), or can exploit the cooperation of others (isolates, peripheral actors, blue)	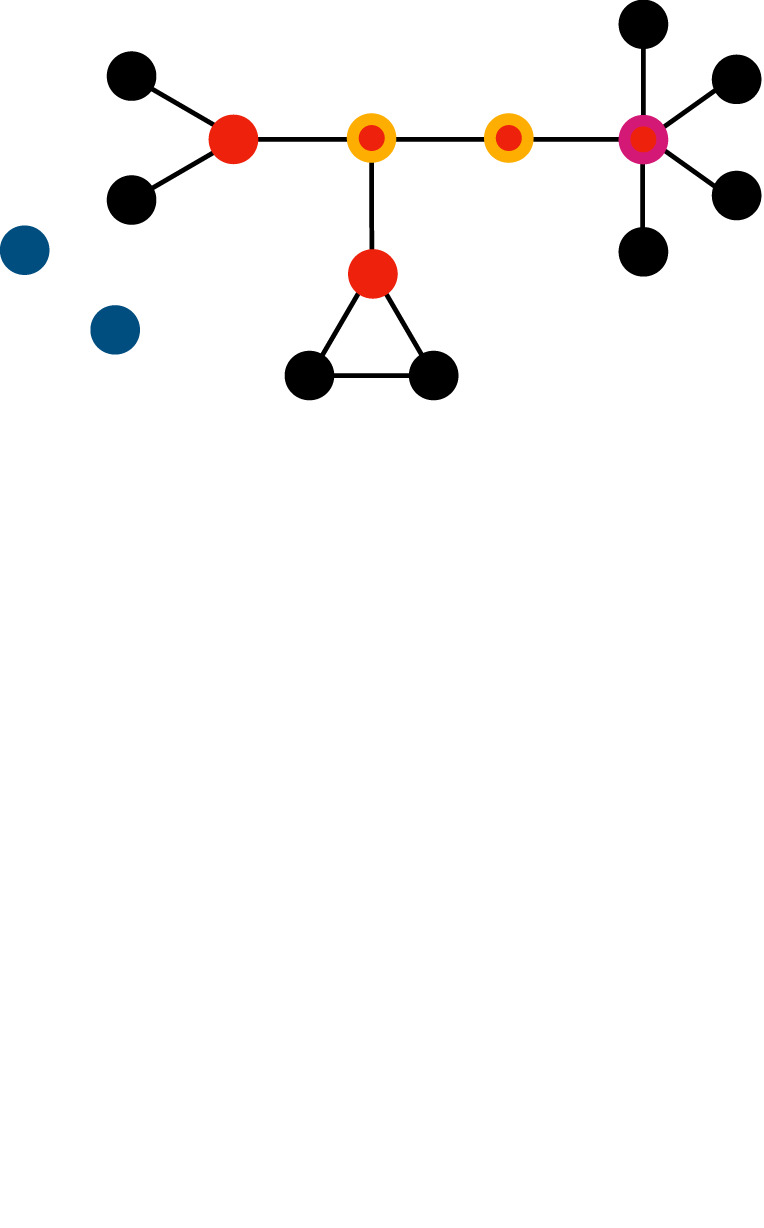
network multiplexity	human networks are multiplex, and networks of interdependence, communication and influence are just partially overlapping	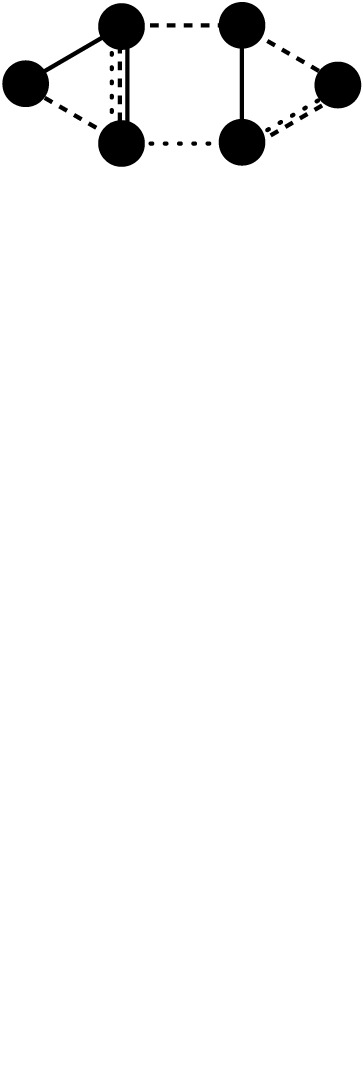
gossip	a sender *i* communicating to a receiver *j* about a target *k* who is absent or unaware of the content [66]	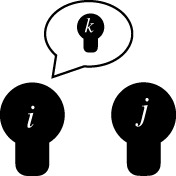
structural constraints on the spread of reputations	the presence of certain network ties (e.g. friendship between the receiver *j* and target *k*, or a 2-path friendship tie of *j–l*–*k*) makes (negative) gossip about *k* by sender *i* to receiver *j* unlikely or forbidden	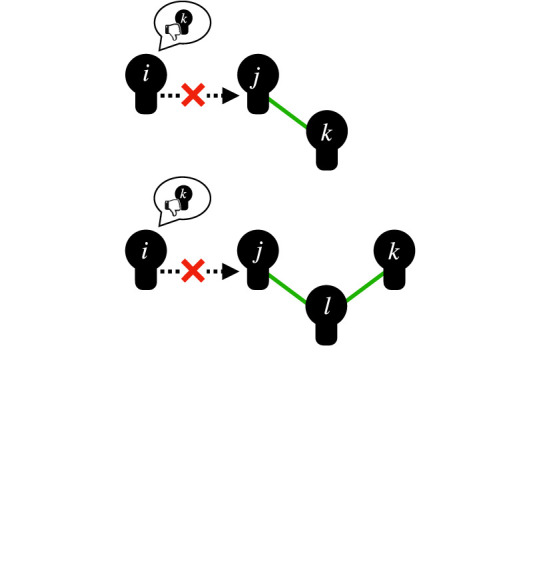
triadic closure/structural holes	triadic closure (left) might be useful to cross-check the validity of reputational information received, while structural holes (right) enable the in-flow of information from independent sources [80]	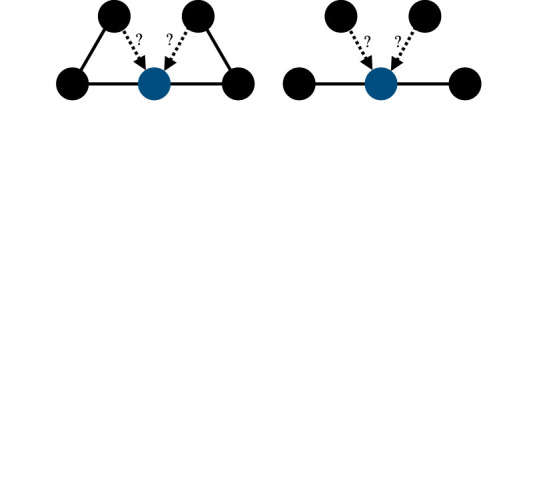
consensual reputation/oppositional cultures	reputational information about an individual may (top) or may not (top versus bottom) be consensual as different subgroups may hold contradicting views about someone's reputation	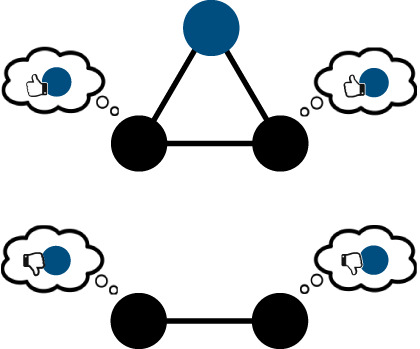

When the likelihood to meet and interact with people remains unchanged over time, the ability to distinguish partners and act according to their previous actions can enable cooperation in networks [81–87]. A simple rule to foster cooperation in networks relates to the ratio between benefits *b*, costs *c* and individual degree *d* (*d* is the number of interaction partners that each agent has in the network). If *b*/*c* > *d*, the evolution of cooperation is theoretically possible without the need of reputation or strategic complexity [88].

People do not all occupy structurally identical positions and social networks often display skewed degree distributions [89]. Degree heterogeneity [90] and scale-free networks in particular have been shown to increase the chances of cooperation [81,84,91–93], though they are also highly vulnerable to error and deletion of nodes [94]. Realistic small-world structures [95] were also found to provide better conditions for cooperation [96]. Systematic investigations of topologies confirmed the superiority of structures in which hubs are integrated in cohesive cliques while they are also linked elsewhere [97]. Structural advantages are due to the benefits of cooperation for hubs and bridging individuals, their larger impact on the behaviour of others, the presence of ties and correlated behaviour among hubs, and high local clustering in small-world networks [98–100]. These results initiated the investigation of degree-based allocation policies that either decrease the required investments or increase the payoffs or aspirations of hubs in order to promote overall cooperation in public good games [100–104]. The strategic positioning of initial cooperators can shorten the time to achieve cooperation, but their placement is non-trivial and depends on the exact game and the network structure [105]. In addition, given their universal presence, highly centralized structures and hierarchical networks have been analysed and the underlying asymmetries found to maintain cooperation in models [106] and in experimental work [107]. Stable hierarchies, however, could imply the lack of motivation and investment in cooperation from lower ranked individuals [108].

Theoretical models that investigated the evolution of cooperation in structured populations, such as in space or in lattices, observed the emergence of cooperation clusters in the population where cooperators meet other cooperators [109–115]. Experimental research, in contrast, suggests that a structured population in itself is not sufficient to solve the problem of cooperation in human groups ([116–119]; see also [120]). The mismatch could be caused by the low benefit to cost ratio in experiments [121], the share and positioning of initial cooperators in the network [105], the individual tendency to cooperate conditionally on the number of cooperative acts of others irrespective of payoff benefits [120] or learning the benefits of free-riding from others leading to a decay of cooperation in any structural setting.

Theoretical work and numerical simulations pinpoint dynamic strategy update rules that can promote cooperation in networks [98,122,123]. Unconditional, proportional or imperfect imitation strategies foster cooperation [109,124] more than innovative strategies, such as the myopic best response rule [125]. Mixing imitative and innovative dynamics is detrimental to cooperation near phase transitions and leads to the downfall of cooperation [126]. A general conclusion is that networks do not support or inhibit cooperation, but their impact depends on the micro-level mechanisms characterized by the strategy update rules individuals employ [124,127]. Results from statistical physics highlight the robust and universal features of phase transitions in problems of cooperation in networks [98,121,122,128,129], the impact of noise [130], mutations [131,132], punishment [129] and quenched distribution of types—which slows down relaxation towards the stationary state extremely [133]—therein.

**Figure 1.**
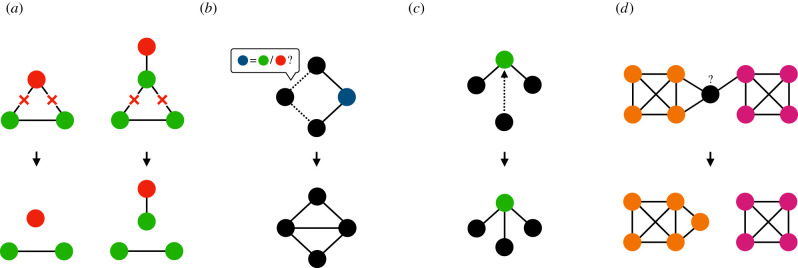
Examples of how network dynamics can relate to cooperation and reputation. (*a*) Breaking relations (crossed ties) can be a form of punishment for defection (left) [134,135] or low reputation (right) [136], which could also originate from cooperation with defectors; (*b*) asking for gossip about future interaction partners might lead to creation of new ties (selection based on access to reliable information); (*c*) preferential attachment to individuals with high reputation (green node); (*d*) cooperation within a cohesive group of individuals might have negative externalities for out-group members, sharpening group boundaries and creating parochial competition [137] which might force bridging individuals (black node) to choose sides.

This brings us to the role of dynamic social networks for the evolution of cooperation. People do not always interact or communicate with an unchanging set of partners. They can attempt to cut ties to previous interaction partners and form new ties (figure 1). This means not only that human interactions are embedded in social networks, but that social networks also change and evolve as a result of social interactions. Theoretical and experimental work has shown that dynamic social networks, labelled also as adaptive networks, in which agents can endogenously influence the network structure by cutting and forming social ties, foster cooperation [94,128,134,138–150] and the positive impact of tie dynamics could even spill over to static parts of the network [151]. The impact of network structure on cooperation depends on the rules and characteristics of dynamic network updates. Cooperation can prevail also in highly unfavourable conditions if strategy adaptation is paired with selective creation of ties [152] or with random creation but selective deletion of ties [153]. Selection of links that ensure higher payoffs in combination with adaptive strategy update offer good chances for cooperation, leading to a hierarchical network [138]. The endogenous development of a strongly heterogeneous topology through mechanisms of growth *and* preferential attachment [89] in which cooperators can secure an advantageous structural position supports cooperation [145,154]. While the role of hubs connected to other hubs is central in this process as their behaviour is imitated with high probability [93,154], cooperators might be located also on nodes of intermediate degree unlike in static networks [155]. The key for the success of cooperation is that the combination of tie updates and strategy updates must ensure that cooperators directly avoid defectors [91,153,156,157] or benefit from a self-organized informal leadership structure [128,138,139,145].

While most models assume that the network of interactions and the network of learning (strategy adoption) are identical and every relation is of equal strength, this is not necessarily the case [98,158,159]. Multiplex networks (cf. table 1) that represent the complex texture of relationships and model numerous layers that represent different social connections help cooperation endure even when the costs for exploitation are high [160]. Endogenous link updating in dynamic multiplex networks could lead to spontaneous symmetry breaking in cooperation levels across the layers [161–163].

The heterogeneity in human exchanges depends on both the diverse social circles people engage in (workplace, family, friends and neighbourhoods) and the strength of the relationships they create. The strength of social ties, meaning the intensity of the relationship and the frequency of communications, acts as a mediator between the maintenance of cooperation and network dynamics: the more robust the links between cooperative people, the more cohesive the cluster of cooperators and the lower the tolerance of defective behaviours [164,165]. That is, through the possibility to choose the interaction partners by strengthening or weakening ties to other agents in a dynamic social network, cooperation can be sustained in both large and small populations [154]. Natural self-organization patterns can dynamically change a social network and induce the spontaneous emergence of cooperative clusters and help populations to become resistant to the invasion of free-riders [146,166,167]. Furthermore, when the network contains both positive and negative ties, network dynamics towards structural balance (e.g. ‘a friend of a friend becomes a friend’ and ‘the enemy of a friend becomes an enemy’) could efficiently drive the network towards in-group cooperation and cohesion [46,168–170].

## 5. Reputation transmission in networks

**Figure 2.**
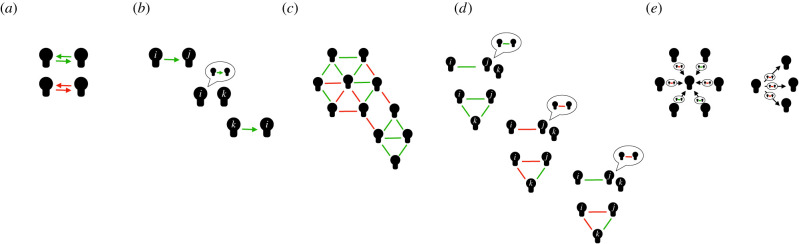
Cooperation in networks and reputation. (*a*) Cooperation can emerge through direct reciprocity in dyadic relationships. (*b*) Individuals can transmit information on past interaction partners to third parties, influencing their behaviour and allowing the evolution of cooperation through indirect reciprocity. (*c*) Often, individuals are engaged in complex social networks with cooperative or uncooperative relationships. (*d*) The transmission of information can influence actions and relationships. Importantly, information in the form of gossip does not need to be correct, allowing strategic (mis)information. The transmission of reputational information through network ties raises the question of how conflicting information from different sources is integrated. (*e*) Agents that have a central position in the network have more channels to transmit information and, hence, may have more influence on the ties of other agents and the evolution of the network.

Beyond the direct relationship between networks and cooperation, reputation-based cooperation is also shaped by networks (figure 2) and networks also change as a result of reputation processes. Reputation affects both cooperation and network formation [120,171].

A useful framework to highlight how reputation is constructed in social networks comes from studies on the diffusion of information in networks [172], from the literature on learning in networks (e.g. [173]) and from network models of opinion dynamics (e.g. [174,175]). From this perspective, reputation in networks can be understood as arising from social influence, that is, as resulting from the communication between people that reinforces each other's views. A ‘shared evaluation’, as opposed to knowledge sharing, likely contains less certainty and requires more social proof, such as receiving the same information from multiple sources. This implies that for reputational information to get transmitted to others, a single source may not be sufficient [80]. Network models of reputation have been proposed to evaluate the reliability of multiple information sources [176–178]. In contrast with models on contagious diseases and information in general, which spread only by contact (a simple contagion), reputational information may be a ‘complex contagion’, i.e. it requires confirmation from multiple sources to be believed and potentially acted upon [172;179, p. 35].

This means that an exposure to reputational information does not immediately imply its acceptance and transmission to others; rather it requires multiple sources of confirmation. This is especially true when the source of reputational information is someone socially distant (several links away in the network). Such verification may be particularly important in situations in which those involved may have strategic incentives to misrepresent reputational information (i.e. spread of false gossip). Therefore, networks that are characterized by high triadic closure, clustering and strong ties should facilitate complex contagions better than network structures with structural holes (open triads), low clustering and weak ties [179,180], because the former allow informational cross-checking and updating.

Extrapolating from this approach, it is likely that the transmission of reputational information might differ depending on the relevance and social (network) distance of the individual whose reputation is under discussion (target). Evidence shows that friendship and work ties influence individuals' propensity to engage in gossip [181,182]. Furthermore, the quantity and quality of the information shared shape individuals’ reputation: high gossip activity decrease people's popularity in the network [183], while gossipers acquire a moral reputation when sharing diagnostic and adequate information that helps to identify others as trustworthy or not [184]. Thus, gossip not only influences the reputation of actors in a social network as ‘good’ or ‘bad’ interaction partners. Individuals in a network can also acquire a reputation as trustworthy or untrustworthy information providers. Such meta-reputations may help to avoid the spreading of false gossip and reduce noise. Our empirical knowledge, however, is still limited on how reputation is disseminated in the social network and to what extent contagion is susceptible to noise in various network topologies, and why an individual could have different reputations in different subsets of the population, as well as how individuals' reputations change based on the information they share.

## 6. The interplay of networks, reputation and cooperation

The complex interplay of networks, reputation and cooperation is such that no simple directionality can be assumed, since all three elements influence each other. Consequently, attempts to grasp the dynamic connections between networks and reputation mechanisms and their impact on cooperation so far have been limited. This holds for integrative theoretical agendas in general, and formal modelling efforts in particular. Both have to disentangle not only the multiplex social ties connecting and the reputational information flowing between the actors in the triad (sender, receiver and target), but also their differential effect on each actor's reputation and cooperative behaviour. A major challenge is capturing the multitude and interrelatedness of potential mechanisms through which networks and reputation may affect cooperation (see [16] for a fuller discussion). Here, we review recent efforts at capturing this dynamic interplay, whether with models or by observational work.

### (a) Intragroup contexts

Some agent-based simulations, experimental studies and field studies have ventured into the coevolution of networks, reputations and cooperation. Partner selection plays a key role in these studies. For example, in a model where individuals interact in their local network, cut ties with individuals of low reputation and establish new relations with nearby individuals with the highest reputation (or with a random agent), free-riders get abandoned and stable and high levels of cooperation emerge parallel with a highly cohesive network structure [136]. This line of research follows up on early modelling work on ‘prisoner's dilemma networks' [185,186] and matches with the results of analytical work that analyses equilibria in games on networks [187].

Experiments also found that dynamics of partner updates based on reputational information lead to high assortativity and stable cooperation [147]. The knowledge of the network structure along with reputational information may be a driver of cooperation and imply the emergence of dense and clustered networks [171]. Other studies have found that cooperation levels increase when more frequent partner updating is allowed (e.g. [134,156]). Large-scale online experiments have also attempted to dissect the complex interplay of network dynamics, reputations and cooperation. It has been found that while reputation information is important for partner choice, it might not even be necessary, and cooperation can be sustained by network dynamics alone [27].

It is important to note that these coevolution models show that the boundary between reputation and punishment mechanisms is fuzzy at best. Some interpret indirect reciprocity models as in line with (passive) punishment models, since the exclusion of low-reputation players from future exchanges can be seen as a sanction [136]. This is also seen in experimental models with dynamic networks, in which this mechanism is referred to as ‘out-for-tat’ [185,186].

Field studies portray a less straightforward picture, owing to intertwined processes of social influence and partner selection (table 1, third row), and in particular, the complex coevolution of networks of cooperation and social status [188], or networks, gossip and reputation [189,190]. For example, a series of studies among employees in Dutch organizations found that partner selection strongly depends on three partner characteristics: (i) the degree to which a potential partner has disclosed reputational information about others, i.e. individuals prefer to build ties to those colleagues who have shared negative third-party gossip with them [183], (ii) the power reputation of potential partners, i.e. individuals prefer to build close interpersonal relations with those colleagues whom they deem informally influential [191], and (iii) the degree to which a potential partner actually occupies an influential brokerage position in the informal network [192]. Finally, partner selection is also strongly influenced by self-monitoring capacity of the selecting party [193], with high self-monitors being more likely than low self-monitors to befriend those whom they or others perceive as powerful [191].

### (b) Intergroup contexts

Intergroup contexts further complicate reputation dynamics and its role in establishing cooperation. A group is a bounded collection of interacting individuals who are interdependent to a certain degree [194]. In informal relations, group boundaries can be ambiguous, though they could be well approximated by detecting a relatively high density of network relations within the group and relatively few ties to members outside the group (e.g. [195–198]). Often, a shared identity binds in-group members together, excluding others. Group membership is associated with parochial cooperation, i.e. high in-group cooperation and low out-group cooperation [137,199–202]. These tendencies are supported by various theoretical accounts, such as social identity theory, self-categorization theory, bounded generalized reciprocity and parochial altruism ([203–207]; for an overview of these theories, see [208]). Though several experiments have demonstrated the importance of social identification for in-group favouritism and in-group cooperation [200,201], experiments have also shown that people cooperate more both with in-group and out-group members when their reputation is at stake (e.g. [209–211]). Not only do people earn reputation from their cooperation, but their reputation may also be affected by their group membership and the actions of group members, as group reputation can be formed from the aggregate of individual reputations [38,39,212–214]. Such group reputations do not help to sustain cooperation with out-group members and generally lead to out-group discrimination [38,201].

One benefit of integrating network dynamics and cooperation is that groups do not have to be assumed to be exogenously given in the first place. Rather, models can allow for the dynamic emergence and dissolution of groups (e.g. [215]) and discrimination (e.g. [216]). Gross & De Dreu [46] provide an agent-based model where agents have personal information on others' cooperativeness, gossip and use the reputations learnt heuristically when deciding to cooperate with others. Applying the four reputation heuristics in structural balance theory, they found that groups emerged dynamically and displayed parochial cooperation; whereas reputation-based partner selection enhanced within-group cooperation, it impeded the emergence and stability of system-wide cooperation. Such models demonstrate that groups can emerge through learning, reputation and gossip, and that these constrain cooperation to certain clusters in a network.

These results are generally supported by behavioural experiments in the laboratory (e.g. [37,134]), and in real-world contexts (e.g. [217–219]). Parochial structures, echo chambers and subgroup polarization may arise when networks and reputations evolve endogenously, increasing the likelihood of in-group loyalty, group-exclusive cooperation and intergroup competition. Further modelling and experimental studies are required, which will help elucidate when parochial cooperation becomes entrenched in processes such as group polarization, and when intergroup tolerance and cooperation are sustained.

## 7. Outlook

Reputation and networks provide paths to large-scale cooperation. Much theoretical and empirical research has been dedicated to understanding the role of reputation and networks for cooperation in isolation and apart from each other. Human cooperation, reputation, and network formation are clearly interrelated. Consequently, the complex causal linkages between reputation, cooperation and networks have gained increasing attention in the literature recently. Investigating the interplay of dynamic social networks and reputation information that is (imperfectly) transmitted through gossip is challenging, from both a theoretical and an empirical perspective. What complicates matters is that individuals can have strategic incentives to spread false gossip [69–71], raising the question of how the validity of reputational information is secured in networks. As individuals associate with groups and attribute reputations also based on group stereotypes, group-bounded parochial cooperation could be the result of reputational dynamics. Investigating the complex interplay of social networks and reputation may, however, be fruitful as it could reveal unexpected emerging dynamics that help explain when or why cooperation remains group-bounded, in what situations networks ‘polarize’, or when cooperation may break down even under conditions that should theoretically favour cooperation. Here, we outline further avenues and open questions for future research, with a particular focus on the issue of complexity, which could be addressed by combining different methods, theoretical viewpoints and strengthening interdisciplinary collaborations.

One important open question is what determines the *stability and efficiency of reputations* and how eroding or developing reputations associates with the maintenance of cooperation. While cooperation has been associated with the convergence on consensual reputations, competition for reputation has also been shown to be an important driver of group-beneficial action. These two views are to a certain degree contradicting and could be reconciled in subsequent research. As competitive altruism theory suggests, competition could exhaust individual efforts and investments while the relative reputational positions remain unaltered, resulting in Red Queen dynamics [220] with the positive externality of large-scale cooperation [53,221–223]. At the other extreme, we also have limited knowledge on those network processes that contribute to the maintenance of false reputations, their reinforcement and the maintenance of suboptimal collective outcomes. The many examples of the Emperor's Clothes are illustrated by theoretical and empirical work [224,225], though these studies are more focused on the emergence and persistence of unpopular norms and beliefs than on cooperation.

Cooperative behaviour may also be *simultaneously motivated* by both network structure and reputational concern. Observational studies by behavioural ecologists of food sharing and other forms of cooperative behaviour have broadly found that multiple mechanisms appear to be operating simultaneously (e.g. [226–232]). Generous acts may help both to reinforce particular interpersonal relationships, and to build reputational standing [54,233–236]. Taking an explicit networks perspective, studies of Lamaleran whale hunters [230,231] and of Canadian Inuit [232] found evidence for reputational signalling, reciprocity and clustering. Ready & Power [232] particularly note how norms of giving and of reciprocity can help to entrench those who wield particularly influential network positions and hold political power.

Another issue is relational multiplexity. When considering real social systems, it is nearly impossible to separate communicative acts and communication networks that may spread reputational information from the underlying social relations the same individuals may be involved in. In other words, networks of exchange and networks of information sharing—both of which may foster cooperation—are co-occurring and mutually overlapping. Social relations are generally multiplex, such that the pathways through which reputational content may flow will often be the same ones through which cooperative exchanges occur [160,162]. This entanglement inevitably adds further complexity to the process by which cooperation may be fostered. A multiplex network perspective may offer a unifying framework for further empirical investigations into the study of cooperation, reputation and networks, by defining different layers of ties in the same system, e.g. ‘who cooperates with whom’, ‘who attributes high/low reputation to whom’ and/or ‘who shares a reputation evaluation about a third party with whom’, ‘who is in a certain relationship (e.g. friendship, trust) with whom’, respectively, so that they can be studied simultaneously. Likewise, future research needs to consider the complex realities of group membership: individuals hold multiple identities [237] in multiplex social networks [238]. Appreciating this multiplexity—and the possibility for the same relationships to have both informational and material exchanges—will be crucial for further advancing our understanding of cooperation [238].

For all network and reputation processes in human cooperation, the social context, in particular, variations in the institutional and intergroup settings, matters. Cooperation is not only maintained through mechanisms of relational mobility and reputation, as outlined in this review. Groups also establish sanctioning systems based on implicit or explicit rules and develop norms of reciprocity [239–241], which can be enforced through partner choice and ostracism, revealing a link between institutions and dynamic social networks. The degree to which networks can sustain or undermine cooperation through reputation, therefore, also depends on the institutional context [242], and on the acceptance and stability of informal and formal institutions that safeguard the maintenance of cooperation [243]. The institutional safeguards themselves have developed on the fundamentals of informal networks and reputation through human history [244–247].

Individual differences are also important in understanding the adaptive function of reputation in networks. People differently manage their reputation depending on whether or not they value collective payoffs and the future [14], while there is also inter-individual variation in reputation domains (such as prosociality and competency, [248]), with concomitant effects on cooperation. Actors experience different socialization processes based on characteristics such as gender that influence the networks of exchange and information sharing described above. Although there is some evidence for overarching patterns, such as men are more likely to engage in competitive altruism than women (see [249] for a meta-analysis of sex differences), they might only hold for specific domains. What would also be informative is focusing on differences in status and relationship history within gender (e.g. [250]) or age group (e.g. [251]). Relatedly, research on the detection and recall of reputation and cooperative behaviours will elucidate proximate mechanisms underpinning the processes discussed in this review; see [46,120,252,253] for work on memory effects.

Finally, as our review has shown, empirical work in this field is based on a wide variety of methods. Agent-based simulation can extend analytical theoretical work in highlighting the macro consequences of micro mechanisms and structural dynamics (e.g. [68,254–256]). Laboratory and field experiments can provide tests for simple hypotheses in controlled environments (e.g. [60,120,257,258]). Field experiments can use games that consider the complexity of individual and group relations in real-world settings (see [259]). The analysis of reputation mechanisms in online markets can provide insights into the efficiency of regulatory practices and could be used to test hypotheses on a massive scale (e.g. [260–264]). Historical data and field observations (e.g. [218,235,265–267]) could provide detailed insights on the build-up and functioning of reputation mechanisms for cooperation in unique contexts and could highlight both the universal character and myriad variegations across human societies. This methodological variety of empirical work demonstrates the added value of cross-disciplinarity. Future research is likely to benefit not only from further embracing this *methodological pluralism*, but also from strengthening the field's methodological and empirical foundations through more powerful mixed-method research designs.

## Data accessibility

This article has no additional data.
