# Correction to: ‘Networks of reliable reputations and cooperation: a review’

**DOI:** 10.1098/rstb.2021.0467

**Published:** 2022-01-03

**Authors:** Károly Takács, Jörg Gross, Martina Testori, Srebrenka Letina, Adam R. Kenny, Eleanor A. Power, Rafael P. M. Wittek

*Phil. Trans. R. Soc. B* **376**, 20200297. (Published 4 October 2021). (doi:10.1098/rstb.2020.0297)

Funding information for one author was omitted from the funding statement.

S.L. is part of the relationship programme supported by The Medical Research Council and Scotland's Chief Scientist Office (grant no. MC_UU_00022/3) and with CSO funding of the Relationships programme (grant no. SPHSU18).

This has been updated on the publisher's website.

